# Optimized Conditions for the Extraction of Phenolic Compounds from *Aeginetia indica* L. and Its Potential Biological Applications

**DOI:** 10.3390/molecules29051050

**Published:** 2024-02-28

**Authors:** Nattira On-Nom, Sirinapa Thangsiri, Woorawee Inthachat, Piya Temviriyanukul, Yuraporn Sahasakul, Amornrat Aursalung, Chaowanee Chupeerach, Uthaiwan Suttisansanee

**Affiliations:** Food and Nutrition Academic and Research Cluster, Institute of Nutrition, Mahidol University, Nakhon Pathom 73170, Thailand; nattira.onn@mahidol.ac.th (N.O.-N.); sirinapa.tha@mahidol.ac.th (S.T.); woorawee.int@mahidol.ac.th (W.I.); piya.tem@mahidol.ac.th (P.T.); yuraporn.sah@mahidol.ac.th (Y.S.); amornrat.aur@mahidol.ac.th (A.A.); chaowanee.chu@mahidol.ac.th (C.C.)

**Keywords:** Alzheimer’s disease, antioxidant activities, Box–Behnken design, enzyme inhibition, green extraction, obesity, response surface methodology, sustainable conservation, type II diabetes

## Abstract

*Aeginetia indica* L., a parasitic root in the Orobanchaceae family, is used as a food colorant in traditional Thai desserts. However, scant information is available on its food applications as well as medicinal properties, while overharvesting by the local people has severely depleted wild plant populations. This research, thus, aimed to extract optimized total phenolic content (TPC) in varying extraction conditions using response surface methodology (RSM) and the Box–Behnken design (BBD). Results indicated that an extraction temperature of 90 °C, 80% (*v*/*v*) aqueous ethanol, and 0.5% (*w*/*v*) solid-to-liquid ratio yielded the highest TPC at 129.39 mg gallic acid equivalent (GAE)/g dry weight (DW). Liquid chromatography–electrospray ionization tandem mass spectrometry (LC-ESI-MS/MS) identified the predominant phenolics as apigenin (109.06 mg/100 g extract) and luteolin (35.32 mg/100 g extract) with trace amounts of naringenin and rutin. Under the optimal extraction condition, the plant extract exhibited antioxidant activities of 5620.58 and 641.52 µmol Trolox equivalent (TE)/g DW determined by oxygen radical absorbance capacity (ORAC) and ferric ion reducing antioxidant power (FRAP) assay, while the scavenging capacity of total radicals at 50% (SC_50_) was determined to be 135.50 µg/mL using 2,2-diphenyl-1-picrylhydrazyl (DPPH) radical scavenging assay. The plant extract also exhibited inhibitory activities against the key enzymes relevant to type II diabetes, obesity, and Alzheimer’s disease, suggesting the potential for medicinal applications.

## 1. Introduction

**Figure 1:**
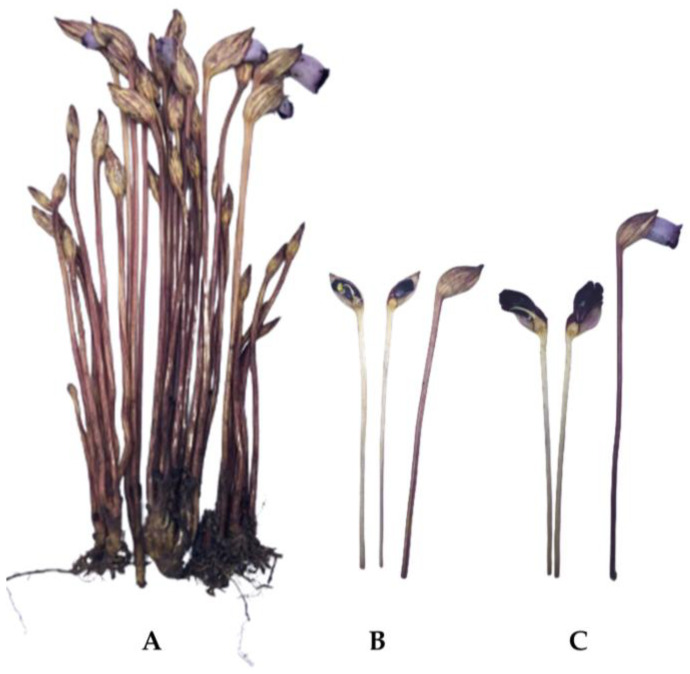
Whole plant of *Aeginetia indica* L. (**A**), sectioned stems and flower buds (**B**), and sectioned stems and fully bloomed flower (**C**).

Non-communicable diseases (NCDs), including cardiovascular diseases, chronic respiratory diseases, diabetes, cancers, and hypertension, are leading global causes of death [1], with annual numbers of fatalities predicted to rise. Many edible plants and herbs have been used as traditional medicines to reduce the risk of NCDs [2]. *Aeginetia indica* L. (also known as “Dok Din” in Thai) is a herbaceous annual plant that is parasitic on roots of rice, sugar cane, and bamboo and is widely distributed throughout China, India, Bangladesh, Myanmar, and other Southeast Asian countries [3]. The plant grows to 15–30 cm high with one to several slender and glabrous peduncles arising from a fleshy rhizome of interwoven roots and purple or purplish-red solitary flowers, which bloom only in the rainy season (September to October in Thailand) [4] (Figure 1). The plant lacks leaves, and its flowers appear suddenly from the ground, with alternative names such as Indian broomrape or forest ghost flower. This parasitic plant is at risk of extinction because it only grows in forest habitats, and no information on reproduction through agricultural management is available.

*A. indica* is used as a food ingredient and coloring agent in traditional Thai desserts by the local people [5] and is also employed as a traditional medicine for dermal swelling and diabetes [6]. Several studies reported on the use of *A. indica* as a tonic and anti-inflammatory medicine in both China and Japan, while seed extracts of *A. indica* exhibited potential anti-tumor activity [7,8,9]. The plant is also used in traditional Taiwanese folk medicine to treat arthritis, coughs, and chronic liver diseases [10], control diabetes in the Philippines [11], reduce fever in Nepal [12], and for urinary tract infection and swelling pain in the throat in China [13]. Scientific evidence has shown the immunological effects of *A. indica* through T cell stimulatory activity [14], while protective effects against paracetamol-induced hepatotoxic and alloxan-induced diabetic mice were also observed [15]. *A. indica* also limited the life cycle of the hepatitis C virus [16] and demonstrated potential applications as an analgesic and antipyretic [3], owing to its bioactive compounds. Preliminary screening determined that *A. indica* contained alkaloids, carbohydrates, glycosides, cardiac glycosides, tannins, terpenoids, saponins, organic acids, reducing sugars, steroids, and phenolics [3,17]. High-performance liquid chromatography (HPLC) has detected the presence of phenolics, including apigenin and its glycoside derivative (apigenin-7-*O*-glucuronide), luteolin and its glycoside derivative (luteolin-7-*O*-glucuronide), anthocyanins (cyanidin-3-*O*-rutinoside and cyanidin-3-*O*-glycoside), quercetin-3-*O*-rutinoside, and naringenin-7-*O*-glucoside, but their contents were not measured [18]. The quantitative identification of other phenolics in *A. indica*, possibly related to its medicinal properties, has also not been investigated. 

Therefore, this research study examined *A. indica* extraction conditions using response surface methodology (RSM) and the Box–Behnken design (BBD) to obtain optimal total phenolic content (TPC). Under the optimal extraction condition, the *A. indica* extract was further investigated for types and quantities of phenolics using liquid chromatography–electrospray ionization tandem mass spectrometry (LC-ESI-MS/MS) with 24 commonly found phenolic standards. In vitro health properties, including antioxidant activities and inhibitions of the key enzymes relevant to type II diabetes (α-amylase, α-glucosidase, and dipeptidyl peptidase-IV (DPP-IV), obesity (lipase), and Alzheimer’s disease (acetylcholinesterase (AChE), butyrylcholinesterase (BChE), and β-secretase (BACE-1)), were also examined. The results provided the appropriate extraction condition to optimize phenolic content and clarified information on the phenolic profiles and preliminary medicinal properties of *A. indica.* The knowledge gained from this research will benefit the promotion of *A. indica* consumption as a functional food ingredient with health properties and also trigger attention and interest to promote plant reproduction through sustainable agricultural management.

## 2. Results

### 2.1. Preliminary Extraction Conditions

The extraction conditions of *A. indica* were investigated using varying ethanol concentration, shaking time, temperature, and solid-to-liquid ratio to examine the ranges of these variables before applying the RSM technique. The first independent variable, ethanol concentration, was examined using controlled variables such as shaking time of 2 h, extraction temperature of 50 °C, and 1% (*w*/*v*) solid-to-liquid ratio (Table 1). Ethanolic extraction is considered safe with low toxicity and environment friendly, while it was previously reported that a mixture of ethanol and water could extract more phenolics than absolute ethanol [19]. Results indicated that TPCs were optimized at 48.72–49.37 mg gallic acid equivalent (GAE)/g dry weight (DW) using 60–80% (*v*/*v*) aqueous ethanol, while the optimal antioxidant activity determined by the ferric ion reducing antioxidant power (FRAP) assay was 184.79 µmol Trolox equivalent (TE)/g DW using 80% (*v*/*v*) aqueous ethanol. Thus, 80% (*v*/*v*) aqueous ethanol concentration was selected for further analysis.

The controlled variables as ethanol concentration 80% (*v*/*v*) aqueous ethanol, temperature 50 °C, and 1% (*w*/*v*) solid-to-liquid ratio were chosen to investigate the effect of shaking time on phenolic extraction conditions (Table 2). Results indicated that TPCs ranging from 41.78 to 42.61 mg GAE/g DW were unaffected by shaking time, while the highest FRAP activity (199.44 µmol TE/g DW) was achieved at a shaking time of 1 h. Thus, this period was selected for further investigations on the effect of temperature on phenolic extraction conditions.

The effect of temperature was examined using the controlled variables of ethanol concentration 80% (*v*/*v*) aqueous ethanol, shaking time 1 h, and 1% (*w*/*v*) solid-to-liquid ratio (Table 3). The optimal TPCs and FRAP activity were 88.56 mg GAE/g DW and 465.74 µmol TE/g DW, respectively, using a temperature of 90 °C. Thus, this extraction temperature was selected for further investigation of solid-to-liquid ratios.

Using the controlled variables of ethanol concentration 80% (*v*/*v*) aqueous ethanol, shaking time 1 h, and temperature 90 °C, the effect of the solid-to-liquid ratio on TPCs and FRAP activities was examined (Table 4). The optimal TPCs were achieved using 1–2% (*w*/*v*) solid-to-liquid ratios, while the highest FRAP activity was recorded at the solid-to-liquid ratio of 1% (*w*/*v*). Thus, this solid-to-liquid ratio was selected for further determination of extraction conditions.

### 2.2. Extraction Conditions by Response Surface Methodology (RSM)

Results in Table 1, Table 2, Table 3 and Table 4 showed that ethanol concentration (Table 1), temperature (Table 3), and solid-to-liquid ratio (Table 4) contributed to the yield of phytochemicals, while extraction time had no effect (Table 2). Thus, the optimal extraction condition was achieved by combining these three factors (ethanol concentration, temperature, and solid-to-liquid ratio) in the BBD.

The independent variables were set at three levels (−1 to 1) as temperature ($X_{1}$; 70, 80, 90 °C), ethanol concentration ($X_{2}$; 60, 80, 100% (*v*/*v*) aqueous ethanol), and solid-to-liquid ratio ($X_{3}$; 0.5, 1.0, 1.5% *w*/*v*), giving 15 randomized experiments to the BBD (Table 5). The experimental TPCs ranged 28.66–129.39 mg GAE/g DW, with the highest detected in $X_{1}$ of 90 °C, $X_{2}\;$ of 80% (*v*/*v*) aqueous ethanol, and $X_{3}$ of 0.5% (*w*/*v*) solid-to-liquid ratio. 

**Figure 2:**
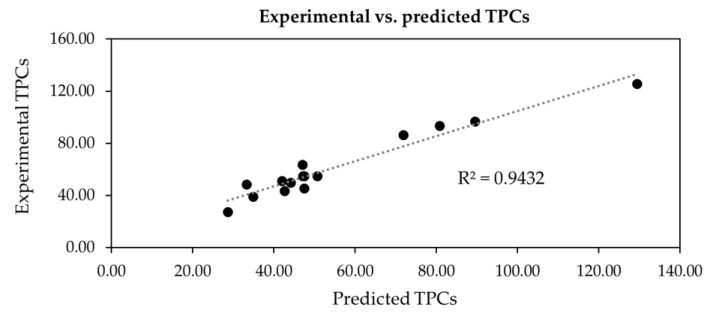
The plot shows the precision between experimental total phenolic contents (TPCs) vs. their predicted values calculated from Equation (1) on different extraction conditions of *Aeginetia indica* L.

In Table 5, the coefficient of determination (R^2^), lack of fit, regression coefficients and *p*-values of the second-order polynomial models for extraction of *A. indica* regarding its TPC were calculated and presented in Table 6. The analysis of variance (ANOVA) indicated that the regression model was significant at *p* < 0.05 (*p*-value = 0.0114), and the lack of fit was not significant. Interestingly, the *p*-value of the monomial coefficient, $X_{1},$ was less than 0.001, indicating a linear relationship between temperature and TPC, and this factor was significant for the extraction of phenolics from *A. indica*. However, the *p*-values of $X_{2}$ and $X_{3}$ were greater than 0.05 (0.4130 and 0.3588, respectively), indicating that the TPC was unaffected by these two monomial coefficients. The *p*-values of the interaction coefficients $X_{1}X_{2},$ $X_{1}X_{3},$ and $X_{2}X_{3}$ were also greater than 0.05 (0.3208, 0.0962, and 0.8623, respectively), indicating that these pairwise interactions did not impact the extraction of phenolics and confirming the sole contribution of extraction temperature. However, the *p*-values of the quadratic coefficients, $X_{1}^{2}$ and $X_{2}^{2},$ were less than 0.01 and 0.05, respectively, indicating their significant influence on phenolic extraction. The *p*-values for R^2^ and adjusted R^2^ were high (0.9452 and 0.8465, respectively), with values close to 1, indicating high TPC prediction validity and reliability. A multiple regression analysis suggested that the relationship between factors of interest and the response variables could be written as a second-order polynomial equation (Equation (1)) as follows:

(1) $$Y=1108.233-34.106X_{1}+3.744X_{2}+\;12.091X_{3}+\;0.029X_{1}X_{2}-0.213X_{1}X_{3}-0.010X_{2}X_{3}+\;0.229X_{1}^{2}-0.038X_{2}^{2}+\;0.250X_{3}^{2},$$

where Y is the predicted TPC (mg GAE/g DW), $X_{1}$ is the temperature (°C), $X_{2}$ is the ethanol concentration (% *v*/*v*), and $X_{3}$ is the solid-to-liquid ratio (% *w*/*v*). The experimental TPC values were compared to the predicted values using Equation (1), with results shown in Table 5 and Figure 2. The R^2^ value of the linear relationship between the experimental and predicted TPC values was 0.9432, in close proximity to 1, confirming the validity and reliability of Equation (1).

**Figure 3:**
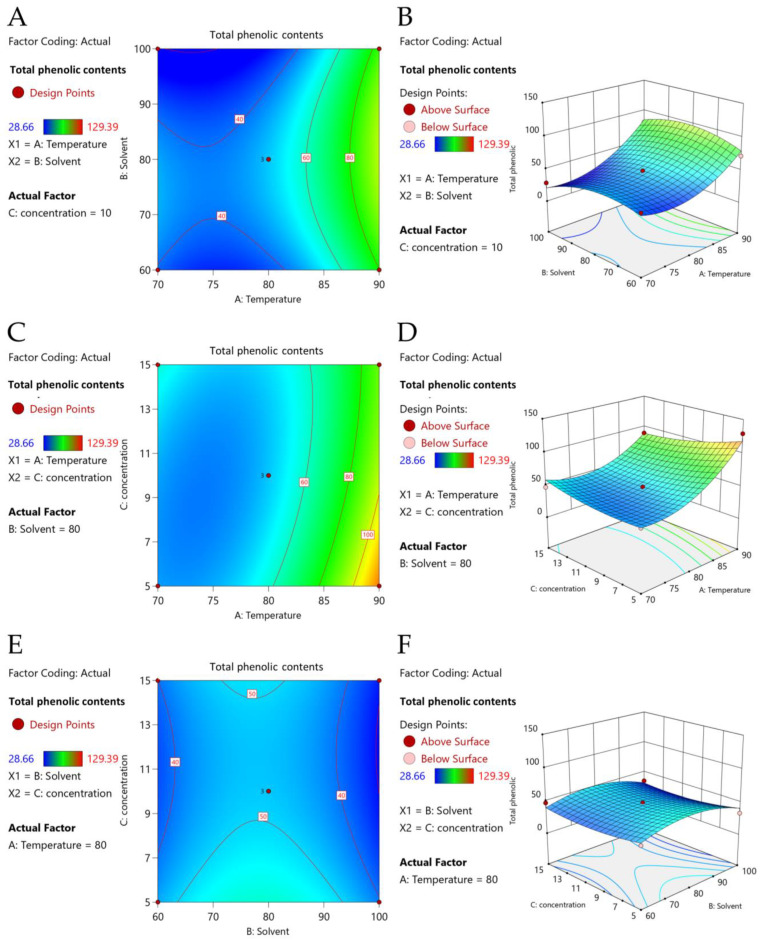
The optimization of *Aeginetia indica* L. using response surface methodology was represented as the contour plots (**A**,**C**,**E**) and response surface plots (**B**,**D**,**F**) of total phenolic contents (TPCs) affected by temperature (X_1_), ethanol concentration (X_2_) and solid-to-liquid ratio (X_3_). Red color indicated high TPCs, while green and blue colors indicated intermediate and low TPCs, respectively.

The strengths of the pairwise interactions between two variables including $X_{1}X_{2}$, $X_{1}X_{3}$, and $X_{2}X_{3}$, where $X_{1}$ is the temperature, $X_{2}$ is the ethanol concentration, and $X_{3}\;$ is the solid-to-liquid ratio, with respect to the response variable, were further examined using RSM and represented as contours and three-dimensional (3D) response surface plots (Figure 3A–F). Results indicated that only temperature had an impact on TPC values, while ethanol concentration and solid-to-liquid ratio had little effect on the extraction of phenolics from *A. indica*. The pairwise effect of temperature and ethanol concentration on TPC was presented as contour and response surface plots of $X_{1}X_{2}$ (Figure 3A,B). Higher temperatures gave increased TPC values, with the highest TPC achieved at a 90 °C temperature. The TPC values increased at higher ethanol concentrations until they reached 82.2% (*v*/*v*) aqueous ethanol and then started to decline. By contrast, ethanol concentration had little effect on TPC. The impacts of temperature and solid-to-liquid ratio on TPC were shown as contour and response surface plots of $X_{1}X_{3}$ (Figure 3C,D), with higher temperatures yielding increased TPC values. The solid-to-liquid ratio had little effect on TPC. The pairwise interaction between ethanol concentration and solid-to-liquid ratio ($X_{2}X_{3}$) on TPC was also shown as contour and response surface plots (Figure 3E,F). The RSM data supported the significance of temperature on TPC, while other factors had little effect.

Conditions for optimal TPC extraction from *A. indica* determined by Design-Expert software (version 13) were 90 °C temperature, 80.2% (*v*/*v*) aqueous ethanol, and 0.5% (*w*/*v*) solid-to-liquid ratio. To generate laboratory reproducible results, these extraction conditions were adjusted to 90 °C temperature, 80% (*v*/*v*) aqueous ethanol, and 0.5% (*w*/*v*) solid-to-liquid ratio. Under these optimal extraction conditions, the predicted TPC was 125.57 mg GAE/g DW and in close proximity to the experimental TPC of 129.39 mg GAE/g DW.

### 2.3. Phytochemical Contents

**Figure 4:**
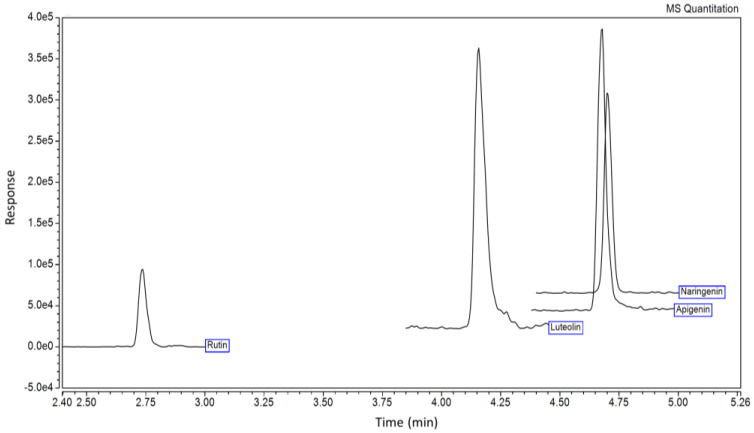
The liquid chromatography–electrospray ionization-tandem mass spectrometry (LC-ESI-MS/MS) chromatogram of *Aeginetia indica* L. extracted under optimized extraction conditions (80% (*v*/*v*) aqueous ethanol, 90 °C extraction temperature, and 0.5% (*w*/*v*) solid-to-liquid ratio).

The phenolic profile of *A. indica* extracted under the optimal extraction conditions was investigated using LC-ESI-MS/MS and compared with 24 authentic phenolic standards with parameters and validations in Supplementary Tables S1 and S2, respectively. Results suggested that the predominant phenolic detected in *A. indica* extract was apigenin 3.1- and 16.6-fold higher than luteolin and naringenin (Figure 4 and Table 7), with only trace amounts of rutin or quercetin-3-*O*-rutinoside detected. The full-scale LC-ESI-MS/MS chromatogram and integration results of the sample are presented in Supplementary Figure S1 and Table S3, respectively. Other than the TPC of 129.41 mg GAE/g DW, the total flavonoid content (TFC) of 64.89 mg quercetin equivalent (QE)/g DW was also detected.

### 2.4. Antioxidant Activities

The antioxidant activities of *A. indica* extracted under the optimal extraction conditions were determined using the oxygen radical absorbance capacity (ORAC), ferric ion reducing antioxidant power (FRAP), and 2,2-diphenyl-1-picrylhydrazyl (DPPH) radical scavenging assays, with results shown in Table 8. The ORAC and FRAP activities were 5620.58 and 641.52 µmol TE/g DW, respectively. The scavenging capacity of total radicals at 50% (SC_50_) was 135.50 µg/mL, as determined by the DPPH radical scavenging assay.

### 2.5. Key Enzyme-Inhibitory Activities

Under the optimal extraction conditions, the preliminarily inhibitory activities of the *A. indica* extract against the key enzymes relevant to type II diabetes (α-amylase, α-glucosidase, and DPP-IV), obesity (lipase), and Alzheimer’s disease (AChE, BChE, and BACE-1) were investigated in comparison to the positive controls (drugs) from our previous works (Table 9). The *A. indica* extract exhibited 35.16% inhibition against α-glucosidase, a non-reducing (1→4)-α-D-glucose terminal hydrolyzing enzyme, using an extract concentration of 0.5 mg/mL, while the inhibition of α-amylase, a (1→4)-α-D-glucoside endohydrolyzing enzyme, was not detected using extract concentrations up to 1 mg/mL. The *A. indica* extract exhibited inhibitory activity against DPP-IV, an enzyme involved in serum glucose homeostasis, with a 26.34% inhibition using an extract concentration of 0.5 mg/mL. Interestingly, inhibition of lipase, a lipid hydrolyzing enzyme, was found to be relatively high at 67.06% using an extract concentration of 1 mg/mL. The *A. indica* extract also inhibited key enzymes in Alzheimer’s disease, including the cholinergic enzymes AChE and BChE and the β-amyloid-producing enzyme BACE-1. The AChE and BChE inhibitions were found to be 38.87 and 21.03%, respectively, using an extract concentration of 1 mg/mL, while only 17.22% inhibition was observed in the BACE-1 assay using an extract concentration of 0. 5 mg/mL.

## 3. Discussion

The parasitic plant *A. indica* is used as a food colorant in traditional Thai dishes, with a historical use as a folk medicine. The plant is consumed as food by the local people, with scant scientific-based information on its therapeutic functions. This research study was the first to investigate the extraction conditions of *A. indica* and optimize TPC. Results indicated that 90 °C extraction temperature, 80% (*v*/*v*) aqueous ethanol, and 0.5% (*w*/*v*) solid-to-liquid ratio gave an optimal TPC of 129.41 mg GAE/g DW. Among the 24 authentic phenolic standards used in the LC-ESI-MS/MS analysis, apigenin was predominantly detected, followed by luteolin, while traces of naringenin and rutin were also found. These phenolics might cause the *A. indica* extract to possess high antioxidant activities and show an inhibition of the key enzymes relevant to type II diabetes (α-amylase, α-glucosidase, and DPP-IV), obesity (lipase), and Alzheimer’s disease (AChE, BChE, and BACE-1).

Different extraction conditions of *A. indica*, including hot-water extraction (decoction) [14,16], 95% (*v*/*v*) ethanolic Soxhlet extraction [14], maceration by either ether, ethyl acetate, ethanol, or water [17], extraction with methanol/acetic acid/water in a ratio of 10/1/10 [18], and 80% (*v*/*v*) methanol extraction [3,15], have been previously performed. However, these extraction conditions were not varied, with no previous studies optimizing extraction conditions to achieve high bioactive compounds. Furthermore, we found that a low solid-to-liquid ratio had higher TPC and antioxidant activities than the ones with elevated solid-to-liquid ratios; the result was also previously reported on the extraction of phenolics from *Terminalia chebula* Retz. fruits [23]. This phenomenon was explained in terms of the mass transfer principle [24], in which interactions between the surface of solid and solvent are increased with a low solid-to-liquid ratio, leading to an increased driving force during the mass transfer of phenolics through plant cell walls into the solvent. RSM and BBD have been employed as effective methods to extract particular bioactive compounds from plant sources [25]. This study is the first to report on a green extraction using RSM and BBD to optimize phenolic extracts from *A. indica*. Under the optimal extraction condition, our TPC value (129.41 mg GAE/g DW) was higher than that extracted with 80% (*v*/*v*) methanol for 14 days (4.5 mg GAE/g DW or 101 mg GAE/g extract) [3]. LC-ESI-MS/MS identified apigenin as predominant, followed by luteolin, naringenin, and rutin (quercetin-3-*O*-rutinoside) in our *A. indica* extract. These phenolics were previously reported but not quantified [18]. Thus, this is the first report to reveal both types and quantities of phenolics in *A. indica* extract. Additionally, due to different extraction procedures, it was previously reported that diterpenes (gibberellins) and phenylpropanoids were extracted from *A. indica* in the range of 0.19–3.83 mg/100 g plant material (or 5.6–115 mg per 3 kg plant material [26]). However, these compounds were less than what we detected in our experiment (i.e., apigenin with 109 mg/100 g plant material). Therefore, diterpenes and phenylpropanoids were not investigated in our experiment. Moreover, we found that the TFC was lower than TFC, suggesting the possibility that the plant extract might contain other phenolics (phenolic acids, tannins, or coumarins) than what we had in the LC-ESI-MS/MS standard list.

These phenolics might contribute to the antioxidant and enzyme-inhibitory activities detected in *A. indica*. A strong correlation between TPC and antioxidant activities was previously reported in many plant extracts [27,28]. However, only the total antioxidant capacity (TAC) measured by the phosphomolybdenum assay (68.3 mg ascorbic acid equivalent/g extract) of *A. indica* was previously reported [3]. Thus, this is the first report to determine antioxidant activities by both the hydrogen atom transfer (HAT)-based ORAC assay and the single electron transfer (SET)-based FRAP and DPPH radical scavenging assays. The HAT-based assay measures the ability of antioxidants to quench free radicals by donating hydrogen atoms, while the SET-based assay measures the antioxidant ability to transfer an electron from a potential antioxidant to any potential electron acceptors. The high antioxidant activity might be a result of the phenolics present in *A. indica* extracts, especially apigenin, which acts as a strong antioxidant owing to its catechol-type structure [29]. A computational analysis of the structural and electronic properties of apigenin indicated that this phenolic followed SET- rather than HAT-based mechanisms [29]. However, our results indicated higher ORAC activity than FRAP and DPPH radical scavenging activities, suggesting that the *A. indica* extract might contain other antioxidants with strong HAT-based action. Moreover, the synergistic effect between bioactive compounds should also be kept in mind, which might be another factor affecting the strength of antioxidants [30].

The high apigenin content might also contribute to in vitro enzyme inhibition by the *A. indica* extract. This extract also inhibited one of the key enzymes involved in type II diabetes, α-glucosidase, while α-amylase inhibitory activity was not detected. Apigenin was reported to reversibly inhibit α-glucosidase activity, with a half maximal inhibitory concentration (IC_50_) of 10.5 µM, in a non-competitive manner [31]. Compared with acarbose, a commercially available α-glucosidase inhibitor with an IC_50_ of 30.4 mM [31], apigenin was considered an effective inhibitor, with α-glucosidase inhibitory activities observed in *A. indica* extract. Apigenin (1 mM) inhibited α-amylase at 5.7% inhibition and was considered a weak inhibitor compared with acarbose, with 81.3% inhibition at the same concentration [32]. The low inhibitory strength of apigenin was a possible explanation for the undetected α-amylase inhibitory activities in the *A. indica* extract. This extract also inhibited DPP-IV, the enzyme that promotes active glucagon-like peptide 1 (GLP-1) content, leading to elevated insulin, decreased glucagon secretions, and lower serum glucose levels. The commercially available DPP-IV inhibitor, sitagliptin, has an IC_50_ value of 4.38 nM [33], and apigenin at a concentration of 0.2 mM inhibited DPP-4 with 46.36% inhibition [34]. The lower strength of apigenin toward DPP-IV inhibition than against α-glucosidase was a possible explanation for why the *A. indica* extract exhibited lower DPP-IV inhibitory activity than α-glucosidase using the same extract concentration. The *A. indica* extract also inhibited lipase, a key enzyme that controls lipid absorption. Compared with orlistat, with an IC_50_ of 0.06 mM, apigenin was considered an effective lipase inhibitor, with an IC_50_ ranging 0.38–0.45 mM in a competitive manner [35,36]. The inhibitory strength of apigenin led to a considerably high lipase inhibition in the *A. indica* extract. In Alzheimer’s disease, the two cholinesterases, AChE and BChE, act as neurotransmitter-degrading enzymes in a cholinergic hypothesis and were also inhibited in the presence of *A. indica* extract. Apigenin exhibited an IC_50_ of 52.9 µM against AChE in a non-competitive manner [37], with a 3.3-fold higher inhibition constant (K_i_) than BChE [38]. The higher AChE inhibitory strength of apigenin than that against BChE might be an explanation for stronger AChE inhibitory activity than against the BChE of *A. indica* extract. The prevention of β-amyloid plaque formation through the inhibition of BACE-1 is also a key concept in the mitigation of the effect of Alzheimer’s disease. Apigenin exhibited an IC_50_ of 38.5 µM against BACE-1 [39] and was shown to inhibit the formation of amyloid plaque by anti-aggregation assays [37]. Thus, it is possible that apigenin could also act as an anti-BACE-1 agent in *A. indica* extracts.

## 4. Materials and Methods

### 4.1. Sample Collection and Preparation

The whole plant of *A. indica* was collected from the Phai Lom village, Non Hom sub-district, Mueang Sakon Nakhon District, Sakon Nakhon Province, Thailand (17°03′07.1″ N 104°11′56.9″ E) on September 2021. The sample was deposited at Sireeruckhachati Nature Learning Park, Mahidol University (Nakhon Pathom, Thailand) and was assigned the voucher specimen number PBM-005746. Dr. Sunisa Sangvirotjanapat (Mahidol University, Nakhon Pathom, Thailand) identified and authenticated the plant according to a reliable reference [40]. 

The sample was cleaned with deionized water, air-dried at room temperature for 2–3 h, and freeze-dried at −50 °C and 0.086 mbar for 3 days using a Heto Powerdry PL9000 freeze dryer (Heto Lab Equipment, Allerod, Denmark). The freeze-dried sample was ground to a fine powder (120 mesh) using a grinder (Phillips 600W series, Phillips Electronics Co., Ltd., Jakarta, Indonesia). The color of the dry sample was determined using a ColorFlex EZ Spectrophotometer (Hunter Associates Laboratory, Reston, VA, USA) and reported in CIELAB units as L* 32.62 ± 0.28, a* 2.20 ± 0.03, and b* 7.37 ± 0.11, where L* represents dark (−) to white (+), a* represents green (−) to red (+), and b* represents blue (−) to yellow (+). The moisture content was determined as 4.67 ± 0.10% using a Halogen HE53 moisture analyzer (Mettler-Toledo AG, Greifensee, Switzerland). The rest of the dry sample was packed in vacuum aluminum foil bags and kept at −20 °C for further analysis.

### 4.2. Optimization of Extraction

The preliminary extraction screening conditions of *A. indica*, such as ethanol concentration, shaking time, temperature, and solid-to-liquid ratio, were varied. Shaking time at 2 h, temperature at 50 °C, and the solid-to-liquid ratio at 1% (*w*/*v*) were investigated for ethanol concentrations ranging 0–100% (*v*/*v*) regarding TPC and FRAP activities (following the methods described in Section 4.3 and Section 4.4). Shaking times of 0.5–6 h were also examined at the fixed conditions of 80% (*v*/*v*) aqueous ethanol, 30 °C temperature, and 1% (*w*/*v*) solid-to-liquid ratio, while temperatures (30–90 °C) were investigated under a fixed shaking time of 1 h, 80% (*v*/*v*) aqueous ethanol, and 1% (*w*/*v*) solid-to-liquid ratio. Lastly, solid-to-liquid ratios of 1–5% (*w*/*v*) were determined under a fixed shaking time of 1 h, 80% (*v*/*v*) aqueous ethanol, and 90 °C temperature.

Using the preliminary extraction conditions that yielded the highest TPC and FRAP activities, the extraction conditions of RSM and BBD were set up using three independent variables with three levels (−1 to 1) of extraction temperature ($X_{1}$; 70, 80, 90 °C), ethanol concentration ($X_{2}$; 60, 80, 100% (*v*/*v*) aqueous ethanol), and solid-to-liquid ratio ($X_{3}$; 0.5, 1.0, 1.5% (*w*/*v*)). The results were used to generate the second-order polynomial equation with coefficient of determination, lack of fit, regression coefficients, and *p*-values of the second-order polynomial models of TPC. A plot comparing the experimental and predicted TPC, as well as contour and response surface plots between the two variables, were also generated.

### 4.3. Analysis of Phenolic Profile

The phenolic profiles of *A. indica* extracted under the optimal extraction condition were analyzed using the LC-ESI-MS/MS technique with previously reported protocols, parameters, and validations [41]. The LC-ESI-MS/MS system consisted of a 2.1 mm × 100 mm, 2.6 μm Accucore RP-MS column (Thermo Fisher Scientific, Bremen, Germany), a 3000 series Dionex Ultimate ultrahigh-performance liquid chromatography (UHPLC) system, a TSQ Quantis Triple Quadrupole mass spectrometer (MS) with multiple MS scanning modes (full scanning and selective reaction monitoring), and a diode array detector. The MS was set to generate negative and positive fragment ions as follows: 50–1000 *m*/*z* mass range, 3500 V positive and negative ions, 30 Arb N_2_ sheath gas, 15 Arb N_2_ auxiliary gas, 350 °C vaporizer, and 325 °C ion transfer tube. The results were analyzed using a Chromeleon 7 chromatography data system (Thermo Fisher Scientific, Bremen, Germany). 

The phenolic standards were sourced from Tokyo Chemical Industry (Tokyo, Japan) and included 3,4-dihydroxybenzoic acid (≥97% T), apigenin (>98.0% HPLC), genistein (>98.0% HPLC), hesperidin (>90.0% HPLC, T), (−)-epigallocatechin gallate (>98.0% HPLC), kaempferol (>97.0% HPLC), 4-hydroxybenzoic acid (>99.0% GC, T), *p*-coumaric acid (>98.0% GC, T), caffeic acid (>98.0% HPLC, T), chlorogenic acid (>98.0% HPLC, T), naringenin (>93.0% HPLC, T), ferulic acid (>98.0% GC, T), sinapic acid (>99.0% GC, T), cinnamic acid (>98.0% HPLC), myricetin (>97.0% HPLC), luteolin (>98.0% HPLC), syringic acid (>97.0% T), and quercetin (>98.0% HPLC, E). Vanillic acid (≥97% HPLC) and rosmarinic acid (≥98% HPLC) were purchased from Sigma-Aldrich (St. Louis, MO, USA), while rutin (≥94% HPLC), gallic acid (97.5–102.5% T), and galangin (≥98.0% HPLC) were bought from Wuhan ChemFaces Biochemical Co., Ltd. (Hubei, China). Isorhamnetin (≥99.0% HPLC) was obtained from Extrasynthese (Genay, France). The parameters and validations of phenolic standards are shown in Supplementary Tables S1 and S2, respectively.

The TPC of *A. indica* extract was also analyzed by Folin-Ciocalteu’s phenol reagent using a previously reported protocol without any modification [19]. Gallic acid at a concentration of 0–200 µg/mL was used to plot a standard curve, with results reported as mg GAE/g DW.

The TFC of the *A. indica* extract was determined using aluminum chloride as a reagent using a previously reported protocol without any modification [42]. Quercetin (0–100 µg/mL) was used to plot a standard curve, with results reported as QE/g DW.

### 4.4. Determination of Biological Properties

The antioxidant potentials of *A. indica* extracted under the optimal extraction condition were determined using ORAC, FRAP, and DPPH radical scavenging assays, as previously reported, without any further modification [19]. Briefly, the kinetic measurement of the ORAC assay using the main reagents as 2,2′-azobis(2-amidinopropane) dihydrochloride and sodium fluorescein, with an excitation wavelength of 485 nm and an emission wavelength of 528 nm. The ORAC activity was calculated using Equation (2).

Area under the curve (AUC) = (0.5 + *f*_1_/*f*_0_ + *f*_2_/*f*_0_ + *f*_3_/*f*_0_ + … + (0.5)*f_i_*/*f*_0_) × CT, (2)

where *f*_0_ is an initial fluorescence at 0 min, *f_i_* is fluorescence at *i* minutes, and CT is the cycle time (min). The FRAP assay employed the main reagents such as 2,4,6-tri(2-pyridyl)-*S*-triazine and FeCl_3_·6H_2_O in acetate buffer and an end-pointed detection wavelength of 600 nm. The FRAP activity was calculated using Equation (3).

(3) $$\mathrm{Antioxidant}\;\mathrm{activity}=\left(\frac{\Delta A-I}{S}\right)\times \;\frac{F}{\mathrm{DW}}\;\times \;\frac{\mathrm{fv}}{1000},$$

where ∆A is the difference in the absorbance of blank and sample, *S* is the slope of a generated standard curve, *I* is the intercept of a generated standard curve, *DW* is the weight of a dry sample (g DW), *F* is the dilution factor, and *f_v_* is the volume of solvent (mL). The DPPH radical scavenging assay used the main reagent as DPPH radical solution and an end-pointed detection wavelength of 520 nm. The DPPH radical scavenging activity was calculated as SC_50_ using Equation (4).

(4) $$\%\;\mathrm{radical}\;\mathrm{scavenging}\;\mathrm{activity}=\left(1-\;\frac{B-b}{A-a}\right)\times 100,$$

where *A* is the absorbance of DPPH reagent and 80% (*v*/*v*) aqueous ethanol (or sample solvent), *a* is the absorbance of 95% (*v*/*v*) aqueous ethanol (or solvent of DPPH reagent) and 80% (*v*/*v*) aqueous ethanol, *B* is the absorbance of DPPH reagent and sample extract, and *b* is the absorbance of 95% (*v*/*v*) aqueous ethanol and sample extract. All assays were performed on a 96-well UV-visible microplate and detected using a Synergy^TM^ HT 96-well UV-visible microplate reader with Gen 5 data analysis software (version 2.09, BioTek Instruments, Inc., Winooski, VT, USA). Trolox was used as a standard, and the results were expressed as µmol TE/g DW for ORAC and FRAP assays, while SC_50_ (µg/mL) was for DPPH radical scavenging assay.

In vitro enzyme inhibitory assays, including α-glucosidase, DPP-IV, lipase, AChE, BChE, and BACE-1, were determined according to the previously reported protocols without any modification [42,43,44,45]. The α-amylase inhibitory assay was performed according to the previous report [43] with change in concentration and volume of enzyme and substrate as follows: 100 µL of 0.06 mg/mL porcine pancreatic α-amylase (type VII, ≥10 unit/mg) and 50 µL of 1 mM 2-chloro-4-nitrophenyl-α-D-maltotrioside. The enzyme inhibitory assays were performed on a Synergy^TM^ HT UV–visible microplate reader, and the results were visualized using Gen 5 data analysis software (BioTek Instruments, Inc., Winooski, VT, USA). The percentage of inhibition (% inhibition) was calculated using Equation (5).

(5) $$\%\;\mathrm{inhibition}=\left(1-\;\frac{B-b}{A-a}\right)\times 100,$$

where *A* is the initial velocity of a reaction (*V*_0_) with an enzyme but without *A. indica* extract (control), *a* is *V*_0_ without an enzyme and *A. indica* extract (control blank), *B* is *V*_0_ of an enzyme and the *A. indica* extract (sample), and *b* is *V*_0_ of the *A. indica* extract but without an enzyme (sample blank). Acarbose was used as a positive control for α-amylase and α-glucosidase inhibitory assays, while saxagliptin was used for the DPP-IV inhibitory assay. Orlistat was used as a positive control for the lipase inhibitory assay, and donepezil was used for the AChE, BChE, and BACE-1 inhibitory assays. All chemicals and reagents were obtained from Sigma-Aldrich (St. Louis, MO, USA).

### 4.5. Statistical Analysis

All the experiments were carried out in triplicate, with results reported as mean ± standard deviation (SD). RSM and BBD were investigated using the Design-Expert software (Stat-Ease Inc., Minneapolis, MN, USA). Significant differences at *p* < 0.05 were analyzed using one-way analysis of variance (ANOVA) and Duncan’s multiple comparison procedure.

## 5. Conclusions

This is the first research paper to report the extraction condition of *A. indica* giving high TPC, type and quantities of extracted phenolics, antioxidant activities determined by both HAT- and SET-based mechanisms, and in vitro inhibitory activities of the key enzymes relevant to obesity, type II diabetes, and Alzheimer’s disease. Under the optimal extraction condition of 90 °C extraction temperature, 80% (*v*/*v*) aqueous ethanol, and 0.5% (*w*/*v*) solid-to-liquid ratio, TPC of 129.41 mg GAE/g DW and TFC of 64.89 mg QE/g DW, as well as ORAC activity of 5620.58 µmol TE/g DW, FRAP activity of 641.52 µmol TE/g DW and DPPH radical scavenging activity with a SC_50_ of 135.50 µg/mL, were detected. The extracted phenolics were predominantly apigenin, followed by luteolin and trace amounts of naringenin and rutin. Preliminary results on enzyme inhibition indicated that *A. indica* extract showed potential for further investigation as an agent for the control of type II diabetes through α-glucosidase inhibition and obesity through lipase inhibition. This information will be of interest to readers in all fields (scientists, food technologists, and horticulturists) as the basis for further investigations of diverse molecular, medicinal, food development, and agricultural management perspectives.

## Figures and Tables

**Table 1:** Effects of different ethanol concentrations of *Aeginetia indica* L. extraction on total phenolic contents (TPCs) and ferric ion reducing antioxidant power (FRAP) activities.

Independent Variable (Ethanol Concentration, % *v*/*v*)	Dependent Variables		Controlled Variables
	TPCs (mg GAE/g DW)	FRAP Activities (µmol TE/g DW)	
0	18.77 ± 0.33 ^e^	43.27 ± 1.49 ^e^	Shaking time 2 h Temperature 50 °C Solid-to-liquid ratio 1% (*w*/*v*)
20	24.48 ± 0.57 ^d^	61.82 ± 3.06 ^d^	
40	40.91 ± 2.18 ^b^	121.70 ± 5.73 ^c^	
60	48.72 ± 0.73 ^a^	161.99 ± 3.71 ^b^	
80	49.37 ± 0.76 ^a^	184.79 ± 7.70 ^a^	
100	35.25 ± 0.43 ^c^	124.29 ± 4.66 ^c^	

All data are expressed as the mean ± standard deviation (SD) of three independent experiments (*n =* 3). Statistical analyses of TPCs and FRAP activities were indicated as a lowercase letter with a significant difference at *p* < 0.05 using one-way analysis of variance (ANOVA) and Duncan’s multiple comparison test. GAE: gallic acid equivalent; DW: dry weight; TE: Trolox equivalent.

**Table 2:** Effects of different shaking time periods of *Aeginetia indica* L. extraction on total phenolic contents (TPCs) and ferric ion reducing antioxidant power (FRAP) activities.

Independent Variable (Shaking Time, h)	Dependent Variables		Controlled Variables
	TPCs (mg GAE/g DW)	FRAP Activities (µmol TE/g DW)	
0.5	42.16 ± 1.75 ^a^	193.24 ± 5.45 ^b^	Solvent 80% (*v*/*v*) ethanol Temperature 50 °C Solid-to-liquid ratio 1% (*w*/*v*)
1	42.40 ± 1.41 ^a^	199.44 ± 3.91 ^a^	
2	41.98 ± 1.61 ^a^	193.81 ± 5.47 ^b^	
4	41.78 ± 1.74 ^a^	193.40 ± 5.59 ^b^	
6	42.61 ± 1.44 ^a^	196.60 ± 6.64 ^ab^	

All data are demonstrated as mean ± standard deviation (SD) of three independent sets of samples analyzed in triplicate (*n* = 3). Statistical analyses of TPCs and FRAP activities were indicated as a lowercase letter with a significant difference at *p* < 0.05 using one-way analysis of variance (ANOVA) and Duncan’s multiple comparison test. GAE: gallic acid equivalent; DW: dry weight; TE: Trolox equivalent.

**Table 3:** Effects of different temperatures of *Aeginetia indica* L. extraction on total phenolic contents (TPCs) and ferric ion reducing antioxidant power (FRAP) activities.

Independent Variable (Temperature, °C)	Dependent Variables		Controlled Variables
	TPCs (mg GAE/g DW)	FRAP Activities (µmol TE/g DW)	
30	45.31 ± 2.32 ^c^	183.03 ± 4.00 ^c^	Shaking time 1 h Solvent 80% (*v*/*v*) ethanol Solid-to-liquid ratio 1% (*w*/*v*)
50	46.52 ± 1.64 ^bc^	191.37 ± 7.05 ^c^	
70	49.10 ± 2.45 ^b^	204.64 ± 7.61 ^b^	
90	88.56 ± 6.24 ^a^	465.74 ± 15.49 ^a^	

All data are demonstrated as mean ± standard deviation (SD) of three independent sets of samples analyzed in triplicate (*n* = 3). Statistical analyses of TPCs and FRAP activities were indicated as a lowercase letter with a significant difference at *p* < 0.05 using one-way analysis of variance (ANOVA) and Duncan’s multiple comparison test. GAE: gallic acid equivalent; DW: dry weight; TE: Trolox equivalent.

**Table 4:** Effects of different solid-to-liquid ratios of *Aeginetia indica* L. extraction on total phenolic contents (TPCs) and ferric ion reducing antioxidant power (FRAP) activities.

Independent Variable (Solid-to-Liquid Ratio, % *w*/*v*)	Dependent Variables		Controlled Variables
	TPCs (mg GAE/g DW)	FRAP Activities (µmol TE/g DW)	
1	96.20 ± 7.45 ^a^	445.83 ± 19.64 ^a^	Shaking time 1 h Solvent 80% (*v*/*v*) ethanol Temperature 90 °C
2	91.80 ± 3.20 ^a^	365.38 ± 19.00 ^b^	
3	81.45 ± 2.57 ^b^	349.47 ± 20.19 ^b^	
4	66.97 ± 5.21 ^c^	292.31 ± 17.99 ^c^	
5	50.67 ± 1.41 ^d^	216.63 ± 9.45 ^d^	

All data are demonstrated as the mean ± standard deviation (SD) of three independent sets of samples analyzed in triplicate (*n* = 3). Statistical analyses of TPCs and FRAP activities were indicated as a lowercase letter with a significant difference at *p* < 0.05 using one-way analysis of variance (ANOVA) and Duncan’s multiple comparison test. GAE: gallic acid equivalent; DW: dry weight; TE: Trolox equivalent.

**Table 5:** Coded and uncoded independent variables (temperature, ethanol concentration, and solid-to-liquid ratio) and dependent variable (total phenolic contents, TPCS) of *Aeginetia indica* L. extraction derived from the Box–Behnken design (BBD).

Run	$X_{1}$: Temperature (°C)		$X_{2}$: Ethanol (% *v*/*v*)		$X_{3}$: Solid-to-Liquid Ratio (% *w*/*v*)		TPCs (mg GAE/g DW)	
	Coded	Uncoded	Coded	Uncoded	Coded	Uncoded	Experimental	Predicted
1	0	80	1	100	1	1.5	34.92 ± 1.48 ^h^	38.77
2	0	80	0	80	0	1.0	50.70 ± 1.80 ^e^	54.78
3	−1	70	1	100	0	1.0	28.66 ± 1.28 ^i^	27.12
4	0	80	1	100	−1	0.5	33.26 ± 1.09 ^h^	48.26
5	−1	70	0	80	1	1.5	47.04 ± 1.11 ^f^	63.60
6	0	80	−1	60	−1	0.5	42.01 ± 1.82 ^g^	50.90
7	1	90	1	100	0	1.0	80.86 ± 1.35 ^c^	93.20
8	1	90	−1	60	0	1.0	71.88 ± 3.66 ^d^	86.24
9	0	80	0	80	0	1.0	47.15 ± 1.40 ^f^	54.78
10	0	80	0	80	0	1.0	47.50 ± 1.54 ^f^	54.78
11	−1	70	0	80	−1	0.5	44.17 ± 2.91 ^g^	49.79
12	1	90	0	80	1	1.5	89.58 ± 1.71 ^b^	96.78
13	−1	70	−1	60	0	1.0	42.67 ± 2.41 ^g^	43.36
14	1	90	0	80	−1	0.5	129.39 ± 4.34 ^a^	125.57
15	0	80	−1	60	1	1.5	47.48 ± 47.48 ^f^	45.41

All data are demonstrated as the mean ± standard deviation (SD) of three independent sets of samples analyzed in triplicate (*n* = 3). Statistical analysis of TPCs was indicated as a lowercase letter with a significant difference at *p* < 0.05 using one-way analysis of variance (ANOVA) and Duncan’s multiple comparison test. GAE: gallic acid equivalent; DW: dry weight.

**Table 6:** Coefficient of determination, regression coefficients, and *p*-value of the second-order polynomial models for total phenolic contents (TPCs).

Source	TPCs					
	Sum of Squares	df	Mean Square	F-Value	*p*-Value	Significance
Model	9385.15	9	1042.79	9.58	0.0114	*
X_1_	5469.01	1	5469.01	50.23	0.0009	***
X_2_	86.72	1	86.72	0.7965	0.4130	
X_3_	111.08	1	111.08	1.02	0.3588	
X_1_X_2_	132.14	1	132.14	1.21	0.3208	
X_1_X_3_	455.40	1	455.40	4.18	0.0962	
X_2_X_3_	3.63	1	3.63	0.0333	0.8623	
X_1_^2^	1927.42	1	1927.42	17.70	0.0084	**
X_2_^2^	862.07	1	862.07	7.92	0.0374	*
X_3_^2^	144.12	1	144.12	1.32	0.3020	
Residual	544.44	5	108.89			
Lack of Fit	544.44	3	181.48	18.33	0.0595	ns
Pure Error	0.0000	2	0.0000			
Cor Total	9929.59	14				
R^2^	0.9452					
R^2^ adjusted	0.8465					

Statistical analyses were analyzed using one-way analysis of variance (ANOVA), with * denoting *p* < 0.05, ** denoting *p* < 0.01, and *** denoting *p* < 0.001. X_1_: temperature; X_2_: ethanol concentration; X_3_: solid-to-liquid ratio; ns: not significant.

**Table 7:** Phenolic profile and total phenolic content (TPC) of *Aeginetia indica* L. extracted under optimized extraction conditions (80% (*v*/*v*) aqueous ethanol, 90 °C extraction temperature, and 0.5% (*w*/*v*) solid-to-liquid ratio).

Phenolic Contents	Ion Mass	Parent Ions (*m*/*z*)	SRM Transitions (*m*/*z*) and Collision Energy (*V*)	RF lens (*V*)	Amount (mg/100 g Extract)
**Phenolic profile**					
Rutin	[M + H]	611.20	303.13 (20.80), 465.20 (12.71V)	198	0.80 ± 0.00 ^c^
Luteolin	[M − H]	285.138	197.000 (15.70 V), 161.113 (17.38 V), 133.054 (37.81 V)	241	35.32 ± 2.10 ^b^
Apigenin	[M − H]	269.075	116.863 (34.28 V), 149.071 (25.13 V), 151.131 (25.05 V)	244	109.06 ± 8.13 ^b^
Naringenin	[M + H]	272.938	146.97 (21.01 V), 153.054 (24.42 V), 119.000 (31.28 V)	160	6.58 ± 0.43 ^c^
**Total phenolic content (mg GAE/g DW)**					129.41 ± 3.23
**Total flavonoid content (mg QE/g DW)**					64.89 ± 5.20

All data are demonstrated as the mean ± standard deviation (SD) of three independent sets of samples analyzed in triplicate (*n* = 3). Statistical analysis of TPCs was indicated as a lowercase letter with a significant difference at *p* < 0.05 using one-way analysis of variance (ANOVA) and Duncan’s multiple comparison test. Information on liquid chromatography–electrospray ionization-tandem mass spectrometry (LC-ESI-MS/MS) parameters was received from the previous literature [19,20]. C3GE: cyanidin-3-*O*-glucoside equivalent; DW: dry weight; GAE: gallic acid equivalent; QE: quercetin equivalent; RF: radio frequencies; SRM: selective reaction monitoring.

**Table 8:** Antioxidant potentials of *Aeginetia indica* L. extracted under optimized extraction conditions (80% (*v*/*v*) aqueous ethanol, 90 °C extraction temperature, and 0.5% (*w*/*v*) solid-to-liquid ratio).

Antioxidant Activities	Amount
DPPH radical scavenging activity (SC_50_, µg/mL)	135.50 ± 11.90
FRAP activity (µmol TE/g DW)	641.52 ± 34.81
ORAC activity (µmol TE/g DW)	5620.58 ± 265.87

All data are demonstrated as the mean ± standard deviation (SD) of three independent sets of samples analyzed in triplicate (*n* = 3). DPPH: 2,2-diphenyl-1-picrylhydrazyl; DW: dry weight; FRAP: ferric ion reducing antioxidant power; ORAC: oxygen radical absorbance capacity; SC_50_: 50% scavenging capacity of total radicals; TE: Trolox equivalent.

**Table 9:** In vitro enzyme inhibitory potential of *Aeginetia indica* L. extracted under optimized extraction conditions (80% (*v*/*v*) aqueous ethanol, 90 °C extraction temperature, and 0.5% (*w*/*v*) solid-to-liquid ratio) compared to the positive controls (drugs).

Related Diseases	Key Enzymes	Enzyme Inhibition (% Inhibition)	Positive Controls (IC_50_, µM)
Type II diabetes	α-Amylase ^1^	ND	Acarbose (14.58) *
	α-Glucosidase ^2^	35.16 ± 2.19	Acarbose (0.53) ^$^
	DPP-IV ^2^	26.34 ± 2.33	Saxagliptin (0.27) *
Obesity	Lipase ^1^	67.06 ± 6.05	Orlistat (7.94) *
Alzheimer’s disease	AChE ^1^	38.87 ± 1.87	Donepezil (3.12) ^#^
	BChE ^1^	21.03 ± 1.71	Donepezil (2.14) ^#^
	BACE-1 ^2^	17.22 ± 0.45	Donepezil (1.31) ^#^

All data are demonstrated as the mean ± standard deviation (SD) of three independent sets of samples analyzed in triplicate (*n* = 3). ^1^ Final concentration of the extract = 1 mg/mL; ^2^ final concentration of the extract = 0.5 mg/mL. AChE: acetylcholinesterase; BChE: butyrylcholinesterase; BACE-1: β-secretase; DPP-IV: dipeptidyl peptidase-IV; IC_50_: half maximal inhibitory concentration; ND: not detected. Information on the positive controls was received from * Chupeerach et al., 2022 [20], ^$^ Promyos et al., 2020 [21], and ^#^ Temviriyanukul et al., 2022 [22].

## Data Availability

The data are contained within this article.

## Supplementary Materials

The following supporting information can be downloaded at https://www.mdpi.com/article/10.3390/molecules29051050/s1. Figure S1: The liquid chromatography–electrospray ionization-tandem mass spectrometry (LC-ESI-MS/MS) chromatogram of *Aeginetia indica* L. extracted under optimized extraction conditions (80% (*v*/*v*) aqueous ethanol, 90 °C extraction temperature, and 0.5% (*w*/*v*) solid-to-liquid ratio); Table S1: Fragment ions of twenty-four phenolic standards using liquid chromatography–electrospray ionization tandem mass spectrometry (LC-ESI-MS/MS); Table S2: The validation parameters of 24 phenolic standards using liquid chromatography–electrospray ionization tandem mass spectrometry (LC-ESI-MS/MS); Table S3: The integration results of *Aeginetia indica* L. using liquid chromatography–electrospray ionization tandem mass spectrometry (LC-ESI-MS/MS).
