# Supplementary material for: Optimized Conditions for the Extraction of Phenolic Compounds from Aeginetia indica L. and Its Potential Biological Applications
Source: Molecules. 2024 Feb 28;29(5):1050. doi: 10.3390/molecules29051050 (PMC10935255; doi:10.3390/molecules29051050)
Supplement: Supplementary file 1 [file molecules-29-01050-s001.zip › molecules-2867035-supplementary.pdf]

molecules

Supplementary Materials

Optimized Conditions for the Extraction of Phenolic Compounds from *Aeginetia indica* L. and Its Potential Biological Applications

Nattira On–Nom, Sirinapa Thangsiri, Woorawee Inthachat, Piya Temviriyanukul, Yuraporn Sahasakul, Amornrat Aursalung, Chaowanee Chupeerach and Uthaiwan Suttisansanee *

Food and Nutrition Academic and Research Cluster, Institute of Nutrition, Mahidol University, Salaya, Phuttamonthon, Nakhon Pathom 73170, Thailand; nattira.onn@mahidol.ac.th (N.O); sirinapa.tha@mahidol.ac.th (S.T.); woorawee.int@mahidol.ac.th (W.I.); piya.tem@mahidol.ac.th (P.T.); yuraporn.sah@mahidol.ac.th (Y.S.); amornrate.aur@mahidol.ac.th (A.A.); chaowanee.chu@mahidol.ac.th (C.C)
* Correspondence: uthaiwan.sut@mahidol.ac.th (U.S.)

Figure S1:

The liquid chromatography-electrospray-ionization-tandem mass spectrometry (LC-ESI-MS/MS) chromatogram of *Aeginetia indica* L. extracted under optimized extraction conditions (80% (*v*/*v*) aqueous ethanol, 90 °C extraction temperature, and 0.5% (*w*/*v*) solid-to-liquid ratio).

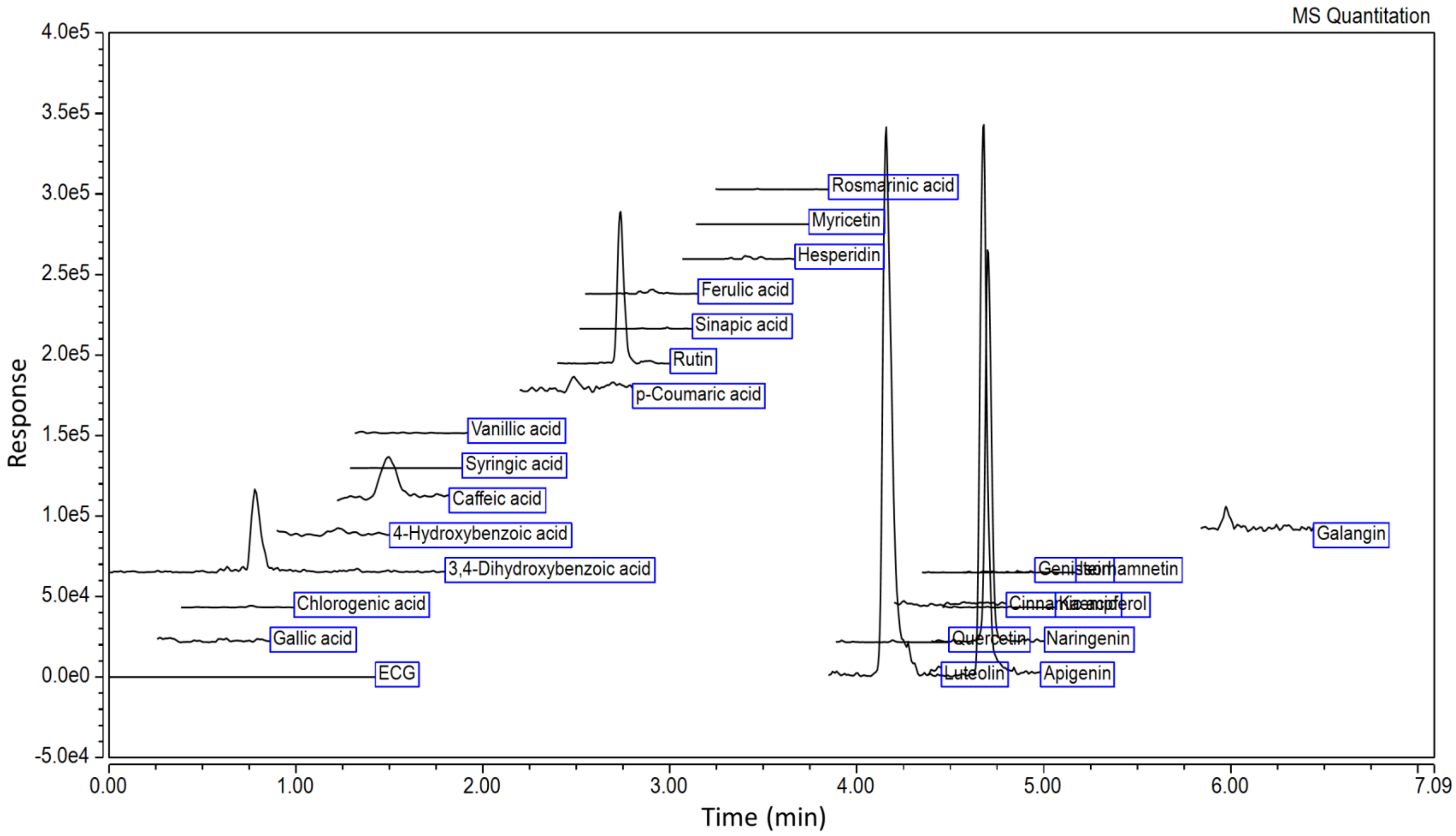

Table S1:

Fragment ions of 24 phenolic standards using liquid chromatography–electrospray ionization–tandem mass spectrometry (LC-ESI-MS/MS).

Compounds	Standards	Ion mass	Parent ions (*m/z*)	SRM transitions (*m/z*) and collision energy (*V*)	*RF lens (V)*
1	Epigallocatechin gallate	[M–H]	457.175	305.155 (16.84 V), 168.97 (17.59 V), 125.042 (40.30 V)	204
2	Gallic acid	[M–H]	169.05	124.988 (14.56 V), 96.917 (18.77 V), 79.185 (22.94 V)	147
3	3,4-Dihydroxybenzoic acid	[M–H]	152.95	109.113 (14.35 V), 81.042 (20.50 V), 91.042 (24.59 V)	128
4	Chlorogenic acid	[M–H]	353.075	179.042 (14.06 V), 191.000 (16.54 V), 85.095 (39.96 V)	148
5	4-Hydroxybenic acid	[M–H]	137.05	92.970 (14.86 V), 65.000 (29.39 V), 75.000 (31.96 V)	110
6	Caffeic acid	[M–H]	179.038	135.054 (15.07 V), 107.071 (22.57 V), 85.042 (31.96 V)	151
7	Syringic acid	[M–H]	197.138	182.185 (13.72 V), 167.113 (19.24 V), 123.095 (22.31 V)	130
8	Vanillic acid	[M–H]	167.000	123.042 (11.66 V), 151.97 (14.59 V), 108.042 (18.65 V)	114
9	*p*-Coumaric acid	[M+H]	165.05	147.054 (11.70 V), 119.113 (19.36 V), 91.125 (25.89 V)	90
10	Rutin	[M+H]	611.20	303.13 (20.80), 465.20 (12.71V)	198
11	Sinapic acid	[M–H]	223.25	208.125 (13.51 V), 164.024 (15.78 V), 192.970 (22.65 V)	141
12	Ferulic acid	[M–H]	192.95	149.125 (11.28 V), 177.970 (13.05 V), 134.042 (16.50 V)	124
13	Hesperidin	[M–H]	609.30	301.179 (24.50 V), 325.179 (27.83 V), 286.125 (41.60 V)	299
14	Myricetin	[M–H]	317.088	178.970 (19.53 V), 150.988 (24.50 V), 137.113 (26.86 V)	245
15	Rosmarinic acid	[M–H]	359.20	197.000 (15.70 V), 161.113 (17.38 V), 133.054 (37.81 V)	175
16	Luteolin	[M–H]	285.138	197.000 (15.70 V), 161.113 (17.38 V), 133.054 (37.81 V)	241
17	Quercetin	[M–H]	301.200	178.976 (18.18 V), 273.125 (19.45 V), 151.042 (21.39 V)	237
18	Cinnamic acid	[M–H]	147.00	103.00 (11.23V), 77.083 (23.07)	107
19	Apigenin	[M–H]	269.075	116.863 (34.28 V), 149.071 (25.13 V), 151.131 (25.05 V)	244
20	Genistein	[M–H]	269.138	224.054 (25.60 V), 159.054 (29.26 V), 132.929 (30.95 V)	239
21	Naringenin	[M+H]	272.938	146.97 (21.01 V), 153.054 (24.42 V), 119.000 (31.28 V)	160

* These data are from the previous literatures [46,47].

Table S1 (Cont.):

Fragment ions of twenty-four phenolic standards using liquid chromatography–electrospray ionization–tandem mass spectrometry (LC-ESI-MS/MS).

Compounds	Standards	Ion mass	Parent ions (*m*/*z*)	*SRM* transitions (*m*/*z*) and collision energy (*V*)	*RF lens (V)*
22	Kaempferol	[M–H]	285.150	184.911 (25.85 V), 239.113 (27.03 V), 186.988 (28.17 V)	260
23	Isorhamnetin	[M–H]	315.088	300.000 (21.30 V), 150.970 (29.14 V), 271.054 (30.57 V)	233
24	Galangin	[M+H]	271.088	165.042 (28.80 V), 197.125 (31.75 V), 153.113 (32.42 V)	248

* These data are from the previous literatures [46,47].

Table S2:

The validation parameters of 24 phenolic standards using liquid chromatography–electrospray ionization–tandem mass spectrometry (LC-ESI-MS/MS).

Compounds	Retention time (min)	Standards	Linear range (µg/mL)	Linear regression equation	Correlation coefficient (R^2)	LOD (µg/mL)	LOQ (µg/mL)	%RSD (Inter-day)	%Recovery		
									Low level (µg/mL)	Medium level (µg/mL)	High level (µg/mL)
1	0.44	Epigallocatechin gallate	0.125–40	y =8533x + 1053.4	0.9985	0.067	0.230	0.023	91.84	85.36	91.37
2	0.564	Gallic acid	0.195–25	y =3323.1x – 2100.4	0.9984	0.04	0.14	0.01	113.05	118.57	109.12
3	0.803	3,4-Dihydroxybenzoic acid	0.195–25	y =11490x – 10877	0.9935	0.010	0.034	0.003	90.59	85.75	89.75
4	0.922	Chlorogenic acid	0.3125–40	y =8377.5x – 3623.5	0.9934	0.017	0.055	0.006	91.94	87.50	95.02
5	1.16	4-Hydroxybenic acid	0.3125–40	y =2482.6x– 3998.4	0.9917	0.027	0.090	0.009	109.67	103.60	101.28
6	1.40	Caffeic acid	0.3125–40	y =12328x–19725	0.9918	0.010	0.035	0.003	105.36	93.98	87.41
7	1.539	Syringic acid	3.125–100	y =68.091x + 230.43	0.9955	0.582	1.939	0.194	116.35	97.42	94.91
8	1.63	Vanillic acid	2.5–100	y =213.67x – 975.72	0.9900	0.15	0.48	0.05	99.86	101.76	100.12
9	2.452	*p*-Coumaric acid	0.3125–40	y =8532.4x–13559	0.9910	0.013	0.042	0.004	88.22	81.36	98.05
10	2.737	Rutin	0.009–1.25	y =49729x – 33.064	0.9999	0.001	0.005	0.0005	94.63	114.00	108.73
11	2.772	Sinapic acid	0.39–25	y =1592.6x – 832.22	0.9977	0.026	0.086	0.009	81.34	92.16	84.22
12	2.851	Ferulic acid	1.56–100	y =559.03x – 1819.2	0.9947	0.155	0.518	0.052	91.51	89.24	93.10
13	3.41	Hesperidin	0.25–40	y =838.63x – 242.2	0.9986	0.07	0.22	0.02	100.43	104.06	108.60
14	3.431	Myricetin	1.25–40	y =303.47x – 601.81	0.9976	0.261	0.871	0.087	113.07	81.77	91.12
15	3.528	Rosmarinic acid	0.3125–40	y =4322.4x-3744.1	0.9956	0.07	0.25	0.02	92.45	106.35	99.62
16	4.158	Luteolin	0.195–12.5	y =8381.9x – 5000.7	0.9945	0.015	0.050	0.0005	84.21	96.21	107.09
17	4.185	Quercetin	0.05–12.5	y =2934x + 917.17	0.9937	0.05	0.18	0.02	83.36	115.06	95.74

* These data are from the previous literatures [46,47].

Table S2 (Cont.):

The validation parameters of 24 phenolic standards using liquid chromatography–electrospray ionization–tandem mass spectrometry (LC-ESI-MS/MS).

Compounds	Retention time (min)	Standards	Linear range (µg/mL)	Linear regression equation	Correlation coefficient (R^2)	LOD (µg/mL)	LOQ (µg/mL)	%RSD (Inter-day)	%Recovery		
									Low level (µg/mL)	Medium level (µg/mL)	High level (µg/mL)
18	4.522	Cinnamic acid	0.039–10	y =6631.9x – 866.59	0.9964	0.049	0.163	0.016	101.94	98.84	95.85
19	4.689	Apigenin	0.34–11	y =1790.7x – 287.7	0.9997	0.127	0.424	0.042	88.84	106.89	114.79
20	4.693	Genistein	0.625–40	y =1247.2x – 1747.1	0.9977	0.049	0.163	0.016	95.33	101.49	11633
21	4.705	Naringenin	0.0008–5	y =16755x + 443.03	0.9932	0.003	0.011	0.001	117.92	96.26	111.08
22	4.79	Kaempferol	0.25–10	y =1006.8x – 346.28	0.9905	0.122	0.406	0.041	92.35	107.69	102.17
23	4.878	Isorhamnetin	0.0098–2.5	y =12698x + 586.16	0.9945	0.016	0.052	0.005	113.57	105.88	111.14
24	6.146	Galangin	0.3125–40	y =5012.1x – 9354.7	0.9879	0.010	0.035	0.003	84.01	112.92	115.80

* These data are from the previous literatures [46,47].

Table S3:

The integration results of *Aeginetia indica* L. using liquid chromatography–electrospray ionization–tandem mass spectrometry (LC-ESI-MS/MS).

No.	Peak Name	Retention Time (min)	Quantitation ion	Area (counts*min)	Height counts	Overall Ion Ratio Confirmation
1	ECG	n.a.	168.97	n.a.	n.a.	Not confirmed
2	Gallic acid	n.a.	124.99	n.a.	n.a.	Not confirmed
3	Chlorogenic acid	0.617	191.00	6	151	Not confirmed
4	3,4-Dihydroxybenzoic acid	0.780	109.11	3418	51454	Not confirmed
5	4-Hydroxybenzoic acid	1.228	92.97	336	4276	Not confirmed
6	Caffeic acid	1.498	135.00	2545	25291	Not confirmed
7	Syringic acid	1.507	182.18	2	41	Not confirmed
8	Vanillic acid	1.642	151.97	16	384	Not confirmed
9	p-Coumaric acid	2.488	147.05	441	8510	Not confirmed
10	Rutin	2.737	303.13	3915	94265	Confirmed
12	Ferulic acid	2.838	134.04	51	2327	Not confirmed
11	Sinapic acid	2.852	208.13	11	371	Not confirmed
13	Hesperidin	3.405	301.18	79	2155	Not confirmed
14	Myricetin	n.a.	150.99	n.a.	n.a.	Not confirmed
15	Rosmarinic acid	3.618	161.11	1	38	Not confirmed
16	Luteolin	4.157	133.07	19312	339531	Confirmed
17	Quercetin	4.188	151.04	34	1297	Not confirmed
18	Cinnamic acid	n.a.	103.00	n.a.	n.a.	Not confirmed
20	Apigenin	4.679	116.86	14872	341696	Confirmed
19	Genistein	4.679	132.93	29	873	Not confirmed
21	Naringenin	4.699	153.05	9299	242991	Confirmed
22	Kaempferol	4.775	239.11	5	315	Not confirmed
23	Isorhamnetin	4.858	300.00	47	1198	Not confirmed
24	Galangin	n.a.	153.11	n.a.	n.a.	Not confirmed

n.a.: not available.
